# Ultralight MOF-Derived Ni_3_S_2_@N, S-Codoped Graphene Aerogels for High-Performance Microwave Absorption

**DOI:** 10.3390/nano12040655

**Published:** 2022-02-16

**Authors:** Wenjing Yu, Bo Liu, Xiaojiao Zhao

**Affiliations:** 1 School of Mathematics and Physics, Jiangsu University of Technology, Changzhou 213001, China; cjsliubo@163.com; 2 Hebei Key Laboratory of Inorganic Nano-Materials, College of Chemistry and Materials Science, Hebei Normal University, Shijiazhuang 050024, China

**Keywords:** graphene aerogels, Ni-MOF, Ni_3_S_2_, microwave absorption

## Abstract

To develop high-performance microwave absorption materials with the features of lightweight, thin thickness, broad bandwidth, and strong absorption, an ultralight Ni_3_S_2_@N, S-codoped graphene aerogel with a density of 13.5 mg/cm^3^ has been fabricated by the use of metal-organic frameworks (MOFs) to directly initiate the gelation of graphene oxide strategy. In such a strategy, dual-functional 1D Ni-MOF nanorods not only act as the gelation agent but also afford the doping elements (N and S) originated from the organic species and the precursor for metal sulfide. Due to the synergistic effects of good impedance matching and multiple losses, the optimal reflection loss (RL) of as-prepared Ni_3_S_2_@N, S-codoped graphene aerogel reaches −46.9 dB at 17.1 GHz with only 2.0 mm and ultralow filling content (1.75 wt%). The maximum effective absorption bandwidth (EAB) reaches 6.3 GHz (11.7–18.0 GHz) at 2.38 mm, covering the whole Ku band. Moreover, the value of EAB with the RL less than −30 dB can be tuned to 12.2 GHz (5.8–18 GHz) at the absorber thickness ranging from 1.9 to 5.0 mm. This work provides insight for rational design and fabrication of multicomponent-containing graphene aerogels, showing the potential application in lightweight and high-performance microwave absorption.

## 1. Introduction

Graphene aerogels (GAs) offer a distinctive combination of high porosity, low density, and tunable conductive properties, which make it grab considerable attention in various applications [1,2,3,4,5], in particular for high-performance electromagnetic (EM) wave -attenuation [6,7,8]. The interconnected porous structures of graphene aerogels can not only provide a sufficient free space inside, improving the impedance match between graphene component and free space but also impart the multiple reflections and scattering of the incident EM waves [7,9,10]. 

Currently, graphene aerogels, either doped with heteroatoms or incorporated with second dielectric loss or magnetization loss materials, have been fabricated to achieve high-performance EM absorbers [6,11]. As an example, the introduction of extra doped atoms can tune the bandgap of reduced graphene oxide and effectively introduce intrinsic defects, breaking the sp^2^ domains in a hexagonal lattice of graphene, improving the conduction loss along with additional polarization relaxation. References [12,13,14] Liu et al. investigated the EM absorption property of N-doped graphene aerogels; the minimal reflection value is −53.9 dB at 3.5 mm with 5 wt% filler loading due to the enhanced dipole relaxation loss from pyrrole/pyridine nitrogen and the conduction loss dominated by graphite nitrogen [15]. On the other hand, designing graphene-based aerogels combined with magnetic materials is also an efficient strategy to achieve improved microwave absorption performance [16,17,18,19]. For instance, a hierarchically porous Fe–Co/N-doped carbon/graphene composite exhibits EAB_max_ of 9.29 GHz, which is attributed to the magnetic-dielectric synergy. 

To further improve the microwave attenuation, the graphene-based aerogels integrated with multiple effects, including conductive loss, dipole polarization, interfacial polarization, magnetic loss, and multiple scattering and reflection have been developed [13,20,21,22,23]. Chen et al. reported a multi-component N-doped graphene aerogel containing N-doped carbon nanotubes and FeNi nanoparticles, which exhibits a strong microwave attenuation (−39.39 dB) of thin thickness (2.0 mm) [21]. Another typical example is multi-dimensional gradient graphene aerogels, comprising 3D carbon nanocoils, 2D graphene sheets, 1D carbon nanofiber, and 0D Fe_3_O_4_@C nanoparticles. This aerogel shows an outstanding minimum RL value of −71.5 dB at 9.5 GHz with a thickness of 2.95 mm, which attributes to multiple loss mechanisms [24]. However, the fabrication of graphene aerogels with a multicomponent usually suffers from problems, including complicated synthetic steps, harsh synthetic conditions, and uncontrolled ratios of each component. Thus, developing a convenient synthetic methodology to fabricate the hierarchically porous aerogels based on graphene oxide and other low-dimensional building blocks is a major challenge. 

Metal-organic framework (MOF) derivatives possess tunable electrical conductivity and abundant terminal groups of carbon matrix, facilitating strong conductive loss and dipole polarization. In addition, MOF derivatives exhibit a prominent feature of high dispersion of nanomaterials into carbon matrix, creating heterogeneous phase interfaces to improve the interfacial polarization loss and optimize the impedance matching [25,26]. 

In this work, a facile and low-cost approach based on 1D Ni-MOF nanorods directly induced gelation strategy has been developed for the preparation of 3D graphene/Ni-MOF aerogels, which are converted to Ni_3_S_2_@N, S-codoped graphene aerogels by further pyrolysis processes. In such a strategy, dual-functional 1D Ni-MOF nanorods not only act as the gelation agent, promoting the gelation of graphene oxide, but also afford the doping elements (N and S) originated from the organic species and the precursor for metal sulfide. The as-prepared Ni_3_S_2_@N, S-codoped graphene aerogels possess satisfactory features, including lightweight, thin thickness, broad bandwidth, and strong absorption. Notably, the reflection loss reaches −46.9 dB at 17.1 GHz with a matching thickness of only 2.0 mm and ultralow filling content (1.75 wt%). The maximum effective absorption bandwidth (EAB_max_) reaches 6.3 GHz (11.7–18.0 GHz) at 2.38 mm, covering the whole Ku band. Therefore, the presented MOF-induced gelation strategy provides an effective pathway to fabricate multicomponent absorbers with a 3D porous structure for achieving excellent EM absorption performance.

## 2. Materials and Methods

### 2.1. Materials

2-Mercaptopyridine, Ni(NO_3_)_2_·6H_2_O, and KOH were obtained from Aladdin Bio-Technology Co., Ltd. (Xi’an, China). Graphene oxide (GO) nanosheets were purchased from XFNANO (www.xfnano.com, accessed on 22 January 2022). 

### 2.2. Preparation of Ni-MOF

In a typical synthesis, 2-Mercaptopyridine (400 mg, 3.6 mmol) and Ni(NO_3_)_2_·6H_2_O (448 mg, 1.8 mmol) were mixed with 16 mL distilled water in a 50 mL Teflon-lined autoclave under stirring until all solid powder dissolved. Subsequently, 236 mg KOH was added 16 mL distilled water to acquire a lucent solution, and then the solution was added dropwise to the above mixture. After stirring for 10 min, the autoclave was transferred to an oven and heated at 75 °C to obtain Ni-MOF nanoparticles. The products were filtered and washed in distilled water and ethanol three times, individually. Finally, the samples were dried in an oven at 80 °C overnight. 

### 2.3. Preparation of Ni_3_S_2_@N,S-Codoped Graphene Aerogels

GO nanosheets and Ni-MOF nanorods were ultrasonically dispersed in cold deionized water for 2 h to obtain 5 mg/mL GO and 5 mg/mL Ni-MOF suspensions, respectively. The Ni-MOF/graphene aerogels were synthesized as follows. Typically, 1 mL Ni-MOF aqueous suspension (5 mg/mL) was dispersed into 2 mL of GO suspension (5 mg/mL) by vigorously shaking for 2 min. The mixed suspension was then transferred into glass bottles, which were heated at 95 °C for 2 h in an oven to obtain Ni-MOF/graphene hydrogels. The hydrogels were frozen for 24 h and freeze-dried for 24 h to obtain Ni-MOF/graphene aerogels. The as-prepared Ni-MOF/graphene aerogels were put into a glass tube and thermally treated at 600, 650, and 700 °C for 1 h in Ar with a heating rate of 3 °C/min to achieve Ni_3_S_2_@N, S-codoped graphene aerogels. The samples are denoted here as Ni_3_S_2_@NSGA-X, where X = 600, 650, and 700, corresponding to the temperatures of thermal treatment.

### 2.4. Characterization 

The microstructure and composition of as-prepared samples were examined by X-ray diffraction (XRD, Rigaku Co., Tokyo, Japan), Raman spectroscopy (LabRam HR Evolution) with a 532 nm laser, X-ray photoelectron spectroscopy (XPS, Thermo Scientific K-Alpha, Waltham, MA, USA), scanning electron microscopy (SEM, Zeiss HD, Jena, Germany) with an energy dispersive X-ray spectroscopy (EDS) system, and transmission electron microscopy (TEM, FEL F20, Lincoln, NE, USA). Electromagnetic parameters were tested by an Agilent PNA-N5244A vector network analyzer (Santa Rosa, CA, USA). The aerogel samples were infiltrated in molten paraffin wax and cut into a standard annulus specimen (Φ_in_: 3.04 mm, Φ_out_: 7.00 mm).

## 3. Results and Discussion

### 3.1. Preparation and Charcteriztion of Ni-MOF

**Figure 1:**
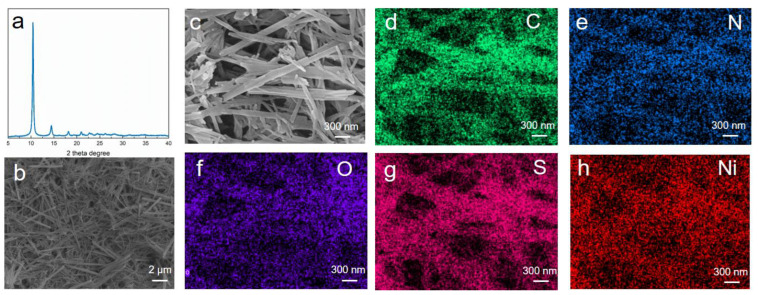
(**a**) XRD pattern (**b**,**c**) SEM and (**d**–**h**) the corresponding EDS mapping images of as-prepared Ni-MOF nanorods.

Ni-MOFs were prepared by the hydrothermal reaction of 2-Mercaptopyridine and Ni(NO_3_)_2_·6H_2_O in a molar ratio of 2:1 in water at 75 °C. Figure 1a shows the XRD pattern of as-prepared Ni-MOF crystals, and the intensive diffraction peaks located at 10.4° and 14.5° are assigned to (002) and (111) planes of Ni-MOF. The SEM image shows that as-prepared Ni-MOFs are present in 1D nanorod morphology (Figure 1b). The EDS mapping images confirm the homogeneous distribution of C, N, O, S, and Ni elements on 1D Ni-MOF nanorods (Figure 1d–h).

### 3.2. Fabrication, Microstructure, and Composition of Ni_3_S_2_@N, S-Codoped Graphene Aerogels

**Figure 2:**
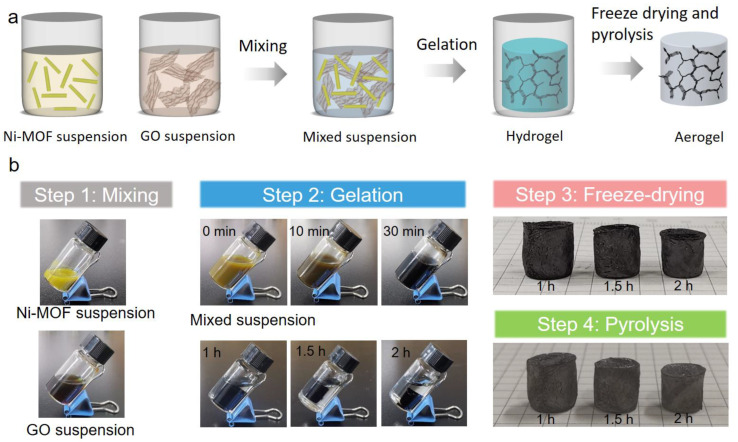
(**a**) Schematic diagram and (**b**) the pictures at each step of the synthetic process for Ni_3_S_2_@N, S-codoped graphene aerogels.

The preparation processes for 3D Ni-MOFs/rGO aerogels and their derived aerogels are schematically illustrated in Figure 2a. Briefly, the GO aqueous suspension and the presynthesized 1D Ni-MOF nanorods are mixed by vigorously shaking. Due to the metal-oxygen covalent or electrostatic interactions between abundant free Ni^2+^ on the surface of 1D Ni-MOF nanorods and oxygenated functional groups (-OH and -COOH) of GO, the GO nanosheets can prevent insoluble 1D Ni-MOF nanorods from precipitation, forming stable suspensions. The mixed suspensions gradually transform into colloids and form stable gels in 1 h under 95 °C. By prolonging the gelation time, the volume of the gels gradually decreases (Figure 2b), indicating that GO could be sufficiently reduced by Ni-MOF without additional chemicals. The ratio of Ni-MOF and GO play a key role in the gelation process of Ni-MOF/rGO hydrogel. A less amount of Ni-MOF will prolong the gelation time, and the resultant gel is brittle due to the fewer sites for cross-linking. If the ratio of Ni-MOF and GO is above 1:1, GO could be sufficiently reduced, and the residual Ni-MOF will be precipitated. Thus, the ratio of Ni-MOF and GO should be optimized to obtain the hydrogels. 

During the further pyrolysis procedure, the N and S elements generated from the deposition of Ni-MOF can be successfully doped into graphene oxide, accompanied by metal sulfide nanoparticles anchored on the surface of graphene oxide. This method benefits from the dual-function of Ni-MOF, in which the organic species afford the doping elements, as well as S being the counterpart of Ni in metal sulfide. The densities of the aerogels pyrolyzed at 650 °C with different gelation times are 8.9, 10.5, and 13.5 mg/cm^3^, respectively, demonstrating the ultralight feature of the Ni_3_S_2_@N, S-codoped graphene aerogels.

**Figure 3:**
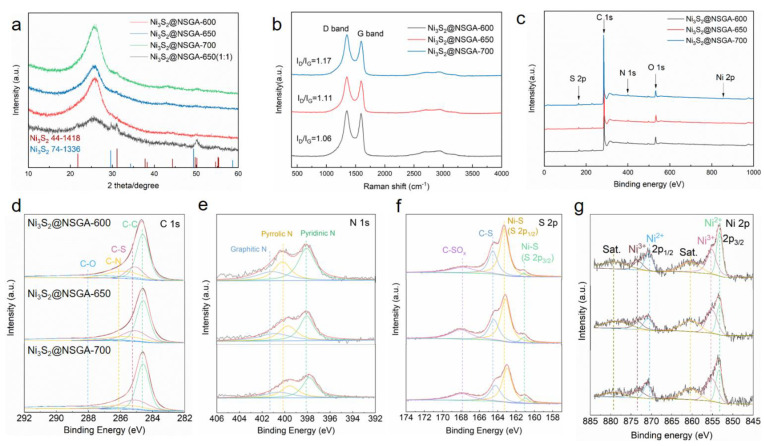
(**a**) XRD patterns, (**b**) Raman spectra, (**c**) XPS survey and high-resolution XPS spectra of (**d**) C 1s, (**e**) N 1s, (**f**) S 2p, (**g**) Ni 2p of as-prepared Ni_3_S_2_@NSGAs.

The crystallographic structures and composition of the prepared aerogels with the gelation time of 2 h were characterized by XRD. Figure 3a shows that all samples exhibit a broad peak at about 25.7°, corresponding to the (002) interlayer of the graphitic structure, implying the reduction of graphene oxide. In addition, the weak diffraction peaks of Ni_3_S_2_ can be detected, especially in the sample with an initial weight ratio of 1:1 of Ni-MOF and graphene oxide, which are well in consistence with XRD patterns of rhombohedral Ni_3_S_2_ (JCPDS No. 44-1418) and cubic Ni_3_S_2_ (JCPDS No. 74-1336) [27]. Figure 3b shows Raman spectra of as-prepared aerogels. Two characteristic peaks located at 1351 and 1594 cm^−1^ are corresponding to the D band and G band, respectively. The intensity ratio of the D band and G band (I_D_/I_G_) gradually increases from 1.06, 1.11 to 1.17 with the increased pyrolysis temperature, indicating that there are much more defects or lattice distortion generated in graphene sheets due to elements doping. This can lead to more defect polarization, which is beneficial to improving the attenuation property of the aerogels.

**Figure 4:**
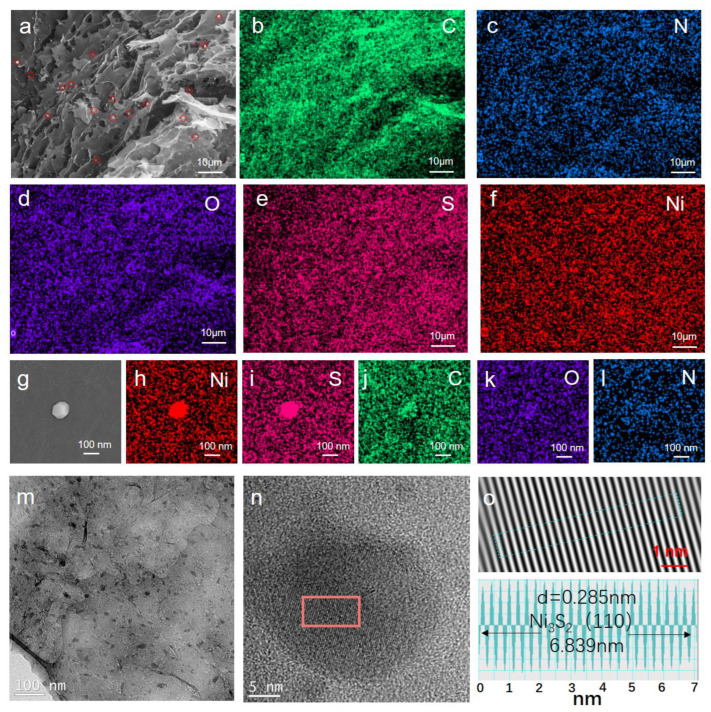
(**a**,**g**) SEM and (**b**–**f**,**h**–**l**) corresponding EDS mapping images, (**m**) TEM, (**n**) HRTEM, and (**o**) IFFT image and the measured interplanar spacing of as-prepared Ni_3_S_2_@NSGA-650.

The chemical composition of as-prepared aerogels is further characterized by XPS. The carbon content (as indicated by the C 1s peaks at ca. 284.6 eV) increases from 86.49, 88.31 to 90.43 at % with the increased pyrolysis temperature. While the intensity of the O 1s peaks located at ca. 532.4 eV continually decreases, the oxygen content declines from 9.23, 7.62 to 5.53 at %, confirming the further reduction of graphene oxide sheets in a high temperature. In addition to C and O elements, N, S, and Ni elements also can be detected in as-prepared aerogels (Figure 3c). The surface elemental composition calculated from XPS was N = 1.84, S = 2.17, and Ni = 0.28 at % of Ni_3_S_2_@NSGA-600. No obvious changes of these elements are found in Ni_3_S_2_@NSGAs prepared at different temperatures. Figure 3d shows the high-resolution C 1s spectra of Ni_3_S_2_@NSGAs including C–C (284.6 eV), C–S (285.1 eV), C–N (285.7 eV), and C–O (288.9 eV) [3,28], respectively. The existence of C–S and C–N bonds proves co-doped N and S elements are in reduced graphene oxide, which are considered as polarized centers, promoting polarization relaxation. Three typical peaks of high-resolution N 1s spectra (Figure 3e) are attributed to pyridinic N, pyrrolic N, and graphitic N, respectively. The binding energy peaks of S 2p_3/2_ and S 2p_1/2_ located at 161.1 and 163.1 eV, respectively, originate from the Ni−S bond. The peak at 164.5 eV is allocated to C−S bonding (Figure 3f). Regarding the XPS spectra of Ni2p (Figure 3g), two peaks at 853.2 and 870.5 eV ascribe to Ni^2+^, while the peaks at 855.3 and 873.4 eV correspond to Ni^3+^ [14,28]. The coexistence of Ni^2+^ and Ni^3+^ in Ni_3_S_2_@NSGAs is beneficial to facilitating the attenuation of microwave energy as the electron hopping from one valent state to another in multivalent ionic materials could induce Debye relaxations. Figure 4a presents the 3D porous structure of the Ni_3_S_2_@NSGA-650 sample, and EDS elemental mappings (Figure 4b–f) reveal that C, N, O, and S atoms are uniformly distributed throughout the N, S-codoped rGO. In addition, the nanoparticles marked by red circles are corresponding to Ni_3_S_2_ over the N, S-codoped rGO surface (Figure 4g–l). The TEM observation shows that numerous nanoparticles with sizes ranging from tens to hundreds of nanometers are embedded into the rGO sheets (Figure 4m). The crystalline lattice spacing of 0.285 nm, consistent with the (110) plane of rhombohedral Ni_3_S_2,_ is confirmed by the high-resolution transmission electron microscope (HRTEM) and inverse fast fourier transition (IFFT) images (Figure 4n,o) [14,28].

### 3.3. EM Absorption Performance of Ni_3_S_2_@N, S-Codoped Graphene Aerogels

Reflection loss (RL) curves, 3D, and 2D representations of the RL for Ni_3_S_2_@NSGAs pyrolyzed at different temperatures are calculated based on the transmission line theory by the following equations:

(1) $$RL=20\mathrm{lg}\left|\frac{Z_{in}-Z_{0}}{Z_{in}+Z_{0}}\right|$$

(2) $$Z_{in}=Z_{0}\sqrt{\frac{\mu_{r}}{\varepsilon_{r}}}\tanh\left(j\frac{2\pi fd\sqrt{\mu_{r}\varepsilon_{r}}}{c}\right)$$

where *Z_in_* is the input characteristic impedance of the absorber, *Z*_0_ stands for the impedance of air, *c* is the velocity of light, *f* is the frequency, and *d* is the absorber thickness.

**Figure 5:**
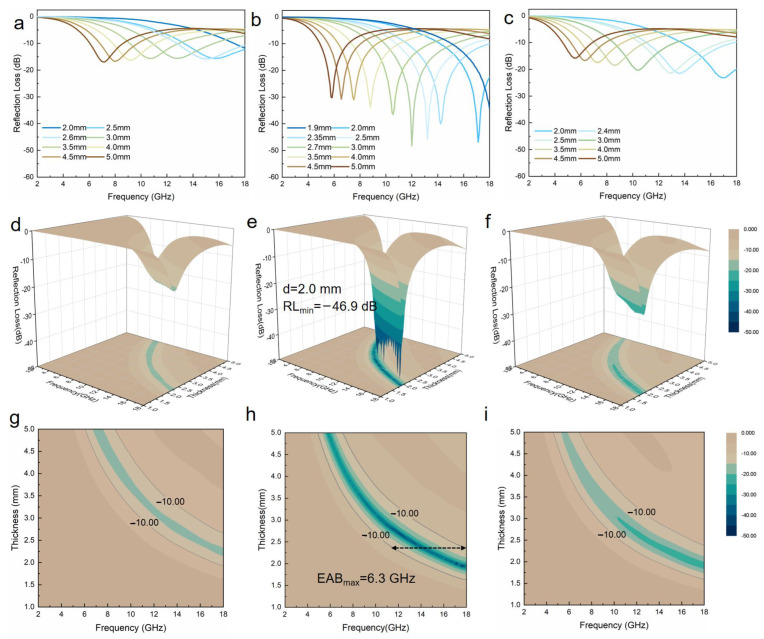
(**a**–**c**) Reflection loss (RL) curves, (**d**–**f**) 3D and (**g**–**i**) 2D representations of the RL for Ni_3_S_2_@NSGAs pyrolyzed at different temperatures.

As shown in Figure 5a–c, all three aerogels can achieve an efficient absorption of −10 dB (90% EM wave absorption), and the maximum effective absorption bandwidth (EAB_max_) values of Ni_3_S_2_@NSGA-600, Ni_3_S_2_@NSGA-650, and Ni_3_S_2_@NSGA-700 can reach 5.3 GHz (12.7–18 GHz), 6.3 GHz (11.7–18 GHz), and 7.0 GHz (11–18 GHz), respectively. Especially, the RL_min_ of Ni_3_S_2_@NSGA-650 reaches −46.9 dB at 17.1 GHz with a thickness of 2.0 mm, and the effective bandwidth is 6.3 GHz at 2.38 mm, covering the whole Ku band (12–18 GHz). Additionally, the effective absorption bandwidth with the RL less than −30 dB can be tuned to 12.2 GHz (5.8–18 GHz) at the absorber thickness of 1.9–5.0 mm, indicating the tunability of the frequency band at different thickness values. The EM performance for Ni_3_S_2_@NSGA-650 is compared with corresponding values in representative recent reports (Table 1). The advantages of ultralow filling content, thin thickness, and broad bandwidth of this ultralight aerogel show its great potential as a new generation of lightweight and high-performance EM wave absorbers.

### 3.4. EM Parameters of Ni_3_S_2_@N, S-Codoped Graphene Aerogels

**Figure 6:**
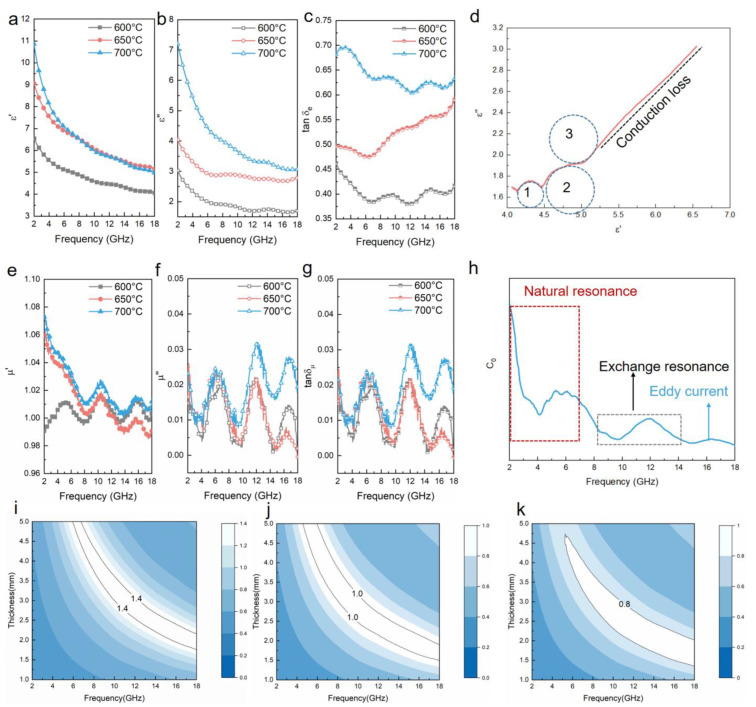
(**a**) Real parts (ε′), (**b**) imaginary parts (ε″) of complex permittivity, (**c**) dielectric loss tangent values (tanδ_ε_), (**e**) real parts (μ′) and (**f**) imaginary parts (μ″) of complex permeability, and (**g**) magnetic loss tangent values (tanδ_μ_) of Ni_3_S_2_@NSGA samples. (**d**) Cole–Cole semicircle curve and (**h**) e C_0_-f curve of Ni_3_S_2_@NSGA-650. (**i**–**k**) 2D contour maps of |Z_in_/Z_0_| at different thicknesses from 2 to 18 GHz for Ni_3_S_2_@NSGA samples.

Due to the introduction of N and S elements into the conjugate structure of rGO, as well as magnetic ingredient Ni_3_S_2_ nanoparticles coupled on the surface of rGO sheets, it can be deduced that both dielectric loss and magnetic loss play a role in EM absorbing behaviors. Accordingly, the parameters of complex permittivity and complex permeability were measured in the frequency range of 2–18 GHz. As shown in Figure 6a, with the increase of frequency, the real permittivity (ε′) values of Ni_3_S_2_@NSGA-600, Ni_3_S_2_@NSGA-650, Ni_3_S_2_@NSGA-700 reduce from 6.54 to 4.06, 9.07 to 5.15 and 10.86 to 4.98, respectively. Meanwhile, the ε″ values of them varied in the range of 1.69–3.03, 2.78–4.04, and 3.05–7.21, respectively(Figure 6b). With the increase in the thermal treatment temperature, the storage (ε′) and dissipation (ε″) capacity of Ni_3_S_2_@NSGA show an upward trend, and Ni_3_S_2_@NSGA-700 presents the best dielectric loss capabilities according to the dielectric dissipation tangent (Figure 6c). In order to explore multiple loss mechanisms in Ni_3_S_2_@NSGA-650, the curves of ε″ versus ε′ were plotted in Figure 6d. The curve can be easily divided into two parts—one is a straight line with larger ε′ related to the conduction loss, which mainly stems from the migration of electrons in a 3D interconnected network structure [22,23]; the other is several semicircles with smaller ε′ with respect to the polarization loss [32,33]. The multiple polarization relaxations in Ni_3_S_2_@NSGA-650 include the dipolar polarizations at the defect and functional groups on the N, S-codoped rGO skeleton, and heterogeneous interfacial polarizations among Ni_3_S_2_ nanoparticles and graphene sheets. 

The complex permeability and magnetic loss tangent of Ni_3_S_2_@NSGA are given in Figure 6e–g. The real permeability (μ′) of Ni_3_S_2_@NSGA-600 fluctuates around 0.99–1.01, while the μ′ values of Ni_3_S_2_@NSGA-650 and Ni_3_S_2_@NSGA-700 exhibit a downward from 1.06 to 0.99 and 1.07 to 1.01 as the frequency increases. Generally, a magnetic loss is attributed to the eddy current loss and magnetic resonance loss (natural resonance and exchange resonance). The eddy current loss can be evaluated by the following equations:

(3) $$\mu^{\prime \prime }\approx \frac{2}{3}\pi \mu_{0}\mu^{\prime }{}^{2}\sigma d^{2}f$$

(4) $$C_{0}=\mu^{\prime \prime }\mu^{\prime }{}^{-2}f^{-1}=\frac{2}{3}\pi \mu_{0}\sigma d^{2}$$

where *μ*_0_ represents vacuum permeability, *C*_0_ is positively correlated with *d^2^* (thickness squared) and *σ* (conductivity). The values of *C*_0_ keep constant in the range of 14–18 GHz in *C*_0_–*f* curves, which indicates that the magnetic loss comes from the eddy current loss. In contrast, the significant vibration area (blue dotted frame) of *C*_0_ at a low frequency (2–7 GHz) shown in Figure 6h, and a slight fluctuation area (red dotted frame) at a high frequency (8–14 GHz) are dominated by natural resonance and exchange resonance, respectively.

Besides the intrinsic microwave attenuation capacity, impedance matching is another key factor for evaluating high-performance absorbing materials [34,35]. The 2D contour maps of Z (Z = |Z_in_/Z_0_|) for Ni_3_S_2_@NSGA samples are presented in Figure 6i–k. Generally, achieving good impedance matching requires that the value of Z is equal to or close to 1. The mismatch of impedance in Ni_3_S_2_@NSGA-600 and Ni_3_S_2_@NSGA-700 is apparent due to that the impedance matching characteristic values of both samples are far away from 1. The Ni_3_S_2_@NSGA-650 possesses the optimal Z values in the whole frequency range. Although it exhibits a moderate attenuation ability, impedance matching allows EM waves to enter into the absorbing materials as much as possible, which is a prerequisite for the Ni_3_S_2_@NSGA-650 aerogel to provide a high EM absorption performance. In the case of Ni_3_S_2_@NSGA-700, though its attenuation ability increases, the missed impedance matching leads to the weaker microwave absorption performance.

### 3.5. EM Absorption Mechanism

Based on the aforementioned discussion, the high-performance microwave absorption property of as-prepared Ni_3_S_2_@NSGA-650 is attributed to the synergistic effects of good impedance matching and multiple losses, which can be revealed from the perspective of the multiscale. (1) Microscopic scale, multiple random reflections, and the scattering of EM waves repeatedly occur at the internal interfaces of the cell walls, leading to the transfer of EM energy to be dissipated as heat, thereby realizing the efficient absorption of EM waves. In addition, the 3D interconnected porous reduced graphene oxide network can establish more conductive paths for electronic transport, endowing the aerogel with high conduction loss. (2) Nanoscale: Ni_3_S_2_ nanoparticle is a typical magnetic loss material, including natural resonance, exchange resonance, and eddy current loss. Despite causing a weak magnetic loss of the material, the low Ni_3_S_2_ content can substantially reduce the effective permittivity of the material, which is favorable for the highly conductive carbon-based absorbers to achieve good impedance matching. The multiple interfaces between Ni_3_S_2_ nanoparticles and reduced graphene oxide nanosheets also enhance the interface polarization. (3) Molecular scale: the introduction of N and S elements into graphene lattice improves the dipolar polarization and the intrinsic defect polarization. The dipolar polarization is ascribed to the different electronegativity between C, N, and S atoms or the residual oxygen functional groups in unsaturated coordination, and the intrinsic defect polarization is resulted from the charge unbalance around the carbon vacancies in the graphene lattice under an alternating electromagnetic field.

## 4. Conclusions

In summary, a high-performance Ni_3_S_2_@N, S-codoped graphene aerogel-based microwave absorber has been fabricated by a facile approach based on 1D Ni-MOF nanorods directly induced gelation strategy. Dual-functional 1D Ni-MOF nanorods not only act as the gelation agent but also afford the doping elements for the generation of N, S-codoped reduced graphene oxide and the precursor for the formation of metal sulfide. The as-prepared Ni_3_S_2_@N, S-codoped graphene aerogel shows a comprehensive absorption performance with an RL_min_ value of −46.9 dB at a thin thickness of 2.0 mm and a broad EAB reaching 6.3 GHz (11.7–18.0 GHz) at 2.38 mm at a low filler loading of 1.75 wt%. It is postulated that the excellent microwave absorption performance is facilitated by the 3D multi-component porous structure, which provides enhanced conductive and polarization loss, magnetic loss, and multiple scattering and reflection. The results found in this work motivate further investigations into multicomponent-containing graphene such as EM absorbers for stealth camouflage techniques.

## Figures and Tables

**Table 1:** Comparison of EM absorption performance between Ni_3_S_2_@NSGA-650 and the reported Ni_3_S_2_-based materials.

EM Wave Absorber	Filler Loading (wt%)	RL_min_ (dB)	EAB_max_ (GHz)	Refs
Ni_3_S_2_@NSGA-650	1.75	−46.9 dB/2.00 mm	6.3 GHz/2.38 mm	This work
H-NSC/Ni_3_S_2_-700	10	−46.80 dB/2.50 mm	7.0 GHz/2.50 mm	[29]
Ni_3_S_2_/C	20	−69.82 dB/2.36 mm	5.56 GHz/1.87 mm	[30]
graphene-NiS/Ni_3_S_2_	50	−55.1 dB/2.70 mm	2.3 GHz/2.70 mm	[31]
n-Ni_x_S_y_@NSC	30	−37.2 dB/1.50 mm	3.84 GHz/1.50 mm	[14]

## Data Availability

The data presented in this study are available on request from the corresponding author.
